# Performance of the aldosterone-to-renin ratio as a screening test for primary aldosteronism in primary care

**DOI:** 10.1007/s12020-022-03084-x

**Published:** 2022-05-27

**Authors:** Joshua Ariens, Andrea R. Horvath, Jun Yang, Kay Weng Choy

**Affiliations:** 1 Northern Clinical School, The University of Melbourne, Melbourne, VIC Australia; 2 Department of Chemical Pathology, NSW Health Pathology, Prince of Wales Hospital, Sydney, NSW Australia; 3 Australian Institute of Health Innovation, Centre for Health Systems and Safety Research, Faculty of Medicine and Health Sciences, Macquarie University, Sydney, NSW Australia; 4 Endocrine Hypertension Group, Hudson Institute of Medical Research, Clayton, VIC Australia; 5 Department of Medicine, Monash University, Clayton, VIC Australia; 6 Department of Endocrinology, Monash Health, Clayton, VIC Australia; 7 Department of Pathology, Northern Health, Epping, VIC Australia

**Keywords:** Primary aldosteronism, Screening, Primary care

## Abstract

Primary aldosteronism (PA) is the most common and potentially curable form of secondary hypertension, affecting 5–10% of primary care patients with hypertension. Primary care physicians have an important role in initiating the screening for PA in patients with hypertension and referring to a specialist service depending on the screening test results. The currently recommended screening test for PA is the plasma aldosterone-to-renin ratio (ARR). Test results are influenced by medications so careful patient preparation is required including adjusting existing antihypertensive medications to avoid diagnostic errors. A range of laboratory method-dependent ARR thresholds are used for the screening of PA around the world. Periodic clinical audits and case reviews by clinicians and the laboratory may help refine the local thresholds. Patients with an abnormally elevated ARR should be referred to a specialist for confirmatory testing while patients with a high pre-test probability but a normal ARR could have a repeat test in view of the within-individual variability. Despite the heterogenous ARR thresholds, measuring the ARR is still more likely to detect PA than not screening at all.

## Background

Primary aldosteronism (PA) is the most common form of secondary hypertension and is characterised by increased adrenal aldosterone secretion and suppressed renin [1]. Aldosterone production in PA is independent of renin and is relatively non-suppressible despite volume expansion and inhibition of angiotensin II [2]. The disease affects 5–10% of patients with hypertension in primary care and up to 30% of those with refractory hypertension [3, 4]. The two most common subtypes of PA are aldosterone-producing adenoma (APA) and bilateral idiopathic hyperaldosteronism (IHA). Compared to blood pressure-matched essential hypertension, patients with PA have an increased risk of stroke, coronary artery disease, and heart failure [5]. Experts have proposed that screening for PA in patients newly diagnosed with hypertension would maximise treatment benefits, minimise end-organ damage and avoid the confounding effects of commonly used antihypertensive medications on screening test results [3, 6]. Hence, general practitioners (GPs) who are at the frontline of hypertension management play a crucial role in screening for PA and interpreting the test results to determine subsequent management [3].

The diagnosis of PA starts with case detection, typically using plasma aldosterone-to-renin ratio (ARR) as the first-line screening test [1]. A normal or elevated plasma aldosterone concentration (PAC) together with a low or suppressed renin concentration is characteristic of PA and gives rise to an elevated ARR [2]. Confirmatory testing is then indicated in most patients with positive screening results to demonstrate the non-suppressibility of aldosterone in the setting of stimuli that would normally suppress aldosterone production, such as salt loading [1]. The current Endocrine Society guideline-recommended confirmatory tests are oral sodium loading test (OSLT), intravenous saline infusion test (SIT), fludrocortisone suppression test (FST) and captopril stimulation test [4]. Once PA is confirmed, patients generally undergo computed tomography (CT) scan of the adrenal glands and adrenal vein sampling for subtyping into unilateral (APA) or bilateral (IHA) PA [1]. The diagnosis of PA provides the clinician with a unique opportunity to offer targeted treatment of the root cause of hypertension or even cure in the case of APA which can be surgically resected [4].

The ARR threshold considered to be abnormal is crucially dependent on the assays used to measure aldosterone and renin. Historically, PAC was analysed by radioimmunoassay (RIA) [7]. The need for increased throughput led to the introduction of chemiluminescent immunoassays (CLIAs) with automation [7]. Marked overestimation of aldosterone by immunoassay can occur in renal impairment due to an accumulation of cross-reacting steroid metabolites [7]. This phenomenon is eliminated by liquid chromatography and tandem mass spectrometry (LC–MS/MS) which specifically measures aldosterone but is not currently widely used given the financial costs and technical expertise required [7]. Renin can be measured based on its enzymatic activity (plasma renin activity, PRA) or on its mass (plasma renin concentration, PRC) [7]. PRA is expressed as the amount of angiotensin I generated per unit of time (e.g., ng/mL/h or nmol/L/h) [7]. PRC is expressed as mU/L [7]. For convenience, automation and speed, many clinical laboratories have switched from PRA to PRC assays.

Hung and colleagues recently published a systematic review on the performance of the ARR as a screening test for PA [8]. Ten studies with a total of 4110 participants were included [8]. The authors found that the clinical performance of ARR varied widely based on the patient population and diagnostic criteria, particularly in terms of sensitivity [8]. It was concluded that no single ARR threshold for interpretation could be recommended [8]. The work by Hung and colleagues is timely but two limitations should be noted. Numerous publications were excluded from the systematic review because they did not perform the confirmatory test on patients who had a ‘negative’ ARR. While this step ensured the accurate calculation of diagnostic performance, it omitted useful information about patients who had a positive ARR and their outcomes following confirmatory testing. Secondly, the majority of the ten studies were from hypertension referral centres [8]. An understanding of the diagnostic performance of the ARR in the primary care setting would be very relevant to GPs who manage the majority of hypertensive patients. The information will also be important for revising guidelines for the screening of PA in primary care. General practitioners could play an important role by actively screening for PA, facilitating early treatment of this readily managed form of secondary hypertension [9]. In a prospective study of detecting PA in Australian primary care, screening people with newly diagnosed hypertension by general practitioners before commencing antihypertensive treatment led to the diagnosis of PA in 14% of screened patients [9].

This current review summarises the performance characteristics of the ARR as a screening test for PA in the primary care setting and aims to answer the questions: what does an ARR result mean for a primary care physician? At what level does it indicate the patient should be referred to a specialist to undergo further testing for PA?

## Methods

The Ovid MEDLINE database was searched for articles relating to primary aldosteronism, ARR and primary care. Additional relevant records were identified through review of the references of selected articles and suggestions from experts in the field. Two reviewers (JA, KWC) were responsible for conducting the search, title and abstract screening, reading articles in full, and data extraction. With regards to screening abstracts, the inclusion criteria were as follows: prospective, retrospective, and cross-sectional observational studies in the primary care settings that include adults aged 18 years or older; studies where the ARR was measured as a screening test for PA and for which guideline-recommended dynamic confirmatory testing for PA was performed.

Key elements of study design, characteristics of the study population, handling of blood pressure medications, aldosterone and renin assays used, threshold for ‘positive’ ARR, type and threshold of dynamic confirmatory testing used were extracted from the included studies.

## Results

**Fig. 1:**
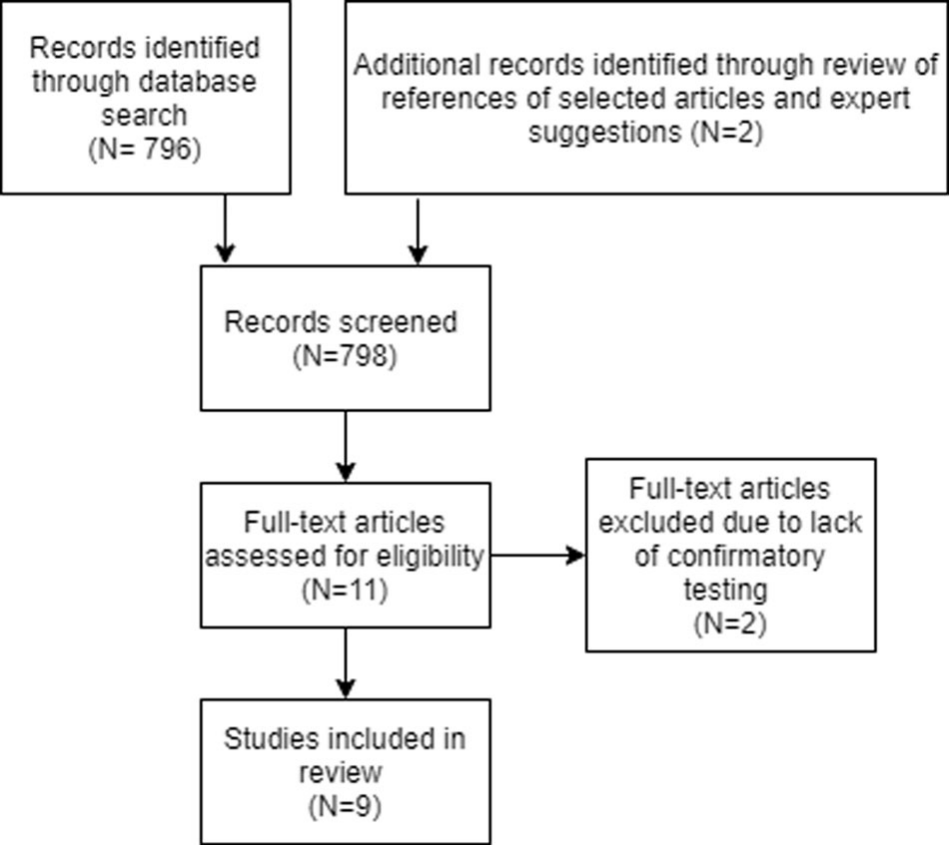
Literature review flow diagram.

Nine studies were included in the review (Fig. 1). The characteristics of included studies are detailed in Table 1. All studies were conducted on patients presenting with hypertension in primary care. The studies spanned the last 21 years encompassing 8180 patients and included publications from the USA [10], Sweden [11–13], China [14], the Netherlands [15], Italy [5], Germany [16], and Singapore [17]. Study population sizes range from 63 to 1672 patients. The most common study population was all patients with hypertension rather than just resistant hypertension.

**Table 1:** Study characteristics and key results

#	Paper, year of publication Study design (prospective or retrospective) Country	Study population (hypertension, resistant hypertension, others)	Inclusion criteria (IC) and exclusion criteria (EC)	Population characteristics Number of patients screened for PA Sex (%M, %F) Age (years) mean (SD or range)	Handling of BP medications	Serum K (mmol/L) (SD or range)	Assays Assay, unit of measurement	% fulfilled screening criteria, % proceeded to confirmatory testing, % tested positive on confirmatory test	ARR threshold	Confirmatory testing (threshold) (if SIT, seated or supine)	Final diagnoses, PA prevalence
1	Galati (2016) [10] Prospective USA	All hypertension	IC: ≥ 18 years, 2x BP > 140/90 or treatment with antihypertensive agents EC: Current use of MRA, systemic steroid, serum creatinine >1.5 mg/dL (>133 µmol/L), previously screened for PA by physicians or already enrolled in another clinical trial	*N* = 296 32%M, 68%F PA cases: 59 (14.1) Controls:59 (14)	Did not discontinue antihypertensive treatment except MRA	PA cases: 3.96 (0.5) Controls: 4.16 (0.4)	Aldosterone: assay not specified, ng/dL Renin: assay not specified, ng/mL/h	14/296 (4.7%) screened positive 6/14 (42.9%) proceeded to confirmatory OSLT 2/6 (33.3%) tested positive on confirmatory test	ARR ≥ 20 ng/dL per ng/mL/h with PAC ≥ 10 ng/dL and suppressed PRA ( < 1 ng/mL/h)	OSLT. PA if post-OSLT U NA > 200 mmol/day and U aldosterone >12 µg/day (or > 33 nmol/day)	PA prevalence, 0.7%
2	Volpe (2013) [11] Prospective Sweden	Newly diagnosed hypertension or had already been treated for SBP > 140 mmHg and/or DBP > 90 mmHg	IC: 18–70 years EC: Secondary hypertension, heart failure, myocardial infarction, transient ischaemic attack/stroke within last 6 months, creatinine >130 µmol/L, malignancy, severe illness, unable to consent to study Excluded 9 patients because of severe illness, cardiovascular disease or renal failure that preclude confirmatory testing	*N* = 178 41%M, 59%F 62 (25–70)	Stopped amiloride and MRA > 4 weeks. If ARR elevated and on beta blocker or non-steroidal anti-inflammatory drug, withdraw for 2 weeks. Given hydralazine, doxazosin, raised dose of calcium antagonist^a^	Majority (93%) had normal K levels Potassium supplement if hypoK	Aldosterone: Siemens Coat-A-Count RIA kit, pmol/L Renin: Electrabox CISBIO immunoradiometric assay, ng/L	6/178 (3.4%) screened positive All 6/6 (100%) proceeded to confirmatory testing 2/6 (33.3%) tested positive on confirmatory test 1/6 had PA confirmed before investigation was started	ARR > 50 pmol/ng (chosen to reduce risk of false negative ARR due to suppressive effects of medications) and PAC > 350 pmol/L.^b^ If ARR elevated, repeated ARR and collect 24 h U aldosterone/NA/K. If U aldosterone elevated (reference interval for urine aldosterone 5.5–35 nmol/24 h), refer for confirmation PA highly unlikely if PRC > 33 ng/L	OSLT (24 h U aldosterone during increased oral salt intake (U NA > 200 mmol/24 h)), or FST. Post-OSLT 24 h U aldosterone threshold and post-FST PAC threshold for PA were not stated	PA prevalence, 1.6% If including 3 more patients with ‘pathological ARR’ but who declined further investigation, PA prevalence, 3.3%
3	Xu (2020) [14] Prospective China	All newly diagnosed hypertension	IC: 18–75 years; new diagnosis (within 12 months) of hypertension, not taking antihypertensives EC: Other causes of secondary hypertension, eGFR <30, severe heart failure New York Heart Association class III	*N* = 1020 65%M, 35%F All: 51 Non-PA: 50 (43–60) Probable PA: 54 (49–66) PA: 47 (35–55)	Severe hypertension: commenced on non-dihydropyridine CCB, terazosin or doxazosin	PA: 3.9 (3.6–4.2)	Aldosterone: DiaSorin LIAISON chemiluminescent immunoassay, ng/dL Renin: DiaSorin LIAISON chemiluminescent immunoassay, mU/L	93/1020 (9.1%) screened positive 61/93 (65.6%) proceeded to confirmatory testing 40/61 (65.6%) tested positive on confirmatory test	ARR > 20 ng/mU (>55 pmol/mU) with PAC >10 ng/dL (>277 pmol/L)	CCT as first test. If post-CCT PAC indeterminate (8–11 ng/dL) then SIT PA if post-CCT PAC > 11 ng/dL or post-SIT PAC > 6 ng/dL	Prevalence, 4% (40/988) PA, 3% probable PA 8 APA, 21 BAH, 11 indeterminate
4	Kayser (2018) [15] Retrospective The Netherlands	Untreated hypertension	IC: Newly diagnosed, untreated hypertension ≥18 years EC: Prior use of antihypertensives, hypertensive crisis, heart failure New York Heart Association class II-IV, eGFR <45, pregnancy, breast feeding, diabetes mellitus, presence of severe comorbidity (seriously interfering with diagnostics or possible therapy)	*N* = 361 52%M, 48%F 53.4 (11.1)	Subjects with prior use of antihypertensives were excluded	PA negative: 4.43 (0.33) PA positive: 4.11 (0.26)	Aldosterone: Siemens Coat-A-Count RIA (Aug 2013 – Dec 2014), pmol/L Beckman Coulter Active ALD RIA (Dec 2014 – Dec 2015), pmol/L Renin: Diagnostic Systems Laboratories DSL-2100 active REN immunoradiometric assay, mU/L	92/361 (35.5%) screened positive 72/92 (78.3%) proceeded to confirmatory testing 9/72 (12.5%) tested positive on confirmatory test	ARR > 40 pmol/mU and PAC >400 pmol/L	Semi-recumbent SIT. PA if post-SIT PAC > 280 pmol/L; PA excluded if post-SIT PAC < 140 pmol/L If post-SIT PAC indeterminate (140–280 pmol/L), SIT repeated; if still indeterminate, PA diagnosis by consensus	Prevalence, 2.6% (9/343)
5	Monticone (2017) [18] Prospective Italy	Newly or previously diagnosed hypertension 19 general practices	IC: 18–60 years, newly or previously diagnosed hypertension EC: Not stated	*N* = 1672 57%M, 43%F Mean age not stated	All interfering medications withdrawn for >4 weeks (6 weeks for diuretics and MRAs) CCB and/or doxazosin used to control BP	HypoK corrected with K supplement	Aldosterone: ng/dL Solid-phase RIA ALDOCTK-2 (DiaSorin) Renin: ng/mL/h RENCTK RIA kit (DiaSorin)	232/1672 (13.9%) screened positive All 232/232 (100%) proceeded to confirmatory testing 99/232 (42.7%) tested positive on confirmatory test	ARR ≥ 30 ng/dL per ng/mL/h) (≥830 pmol/L per ng/mL/h) and PAC >10 ng/dL (>277 pmol/L)	SIT. PA if post-SIT PAC > 5 ng/dL (>139 pmol/L) If contraindication to acute volume expansion, CCT; PA if post-CCT ARR > 30 ng/dL per ng/mL/h	Prevalence, 5.9% (99/1672) Adrenal vein sampling was performed in all patients: 65% BAH, 27% APA, 8% Undetermined
6	Westerdahl (2011) [12] Prospective Sweden	Newly diagnosed, medical untreated hypertension 6 primary health care	IC: Newly diagnosed, medically untreated hypertension EC: Not stated	*N* = 200 43%M, 57%F Female: range, 24–75 years Male: range, 21–75 years	Doxazosin or amlodipine for BP management	PA: 3.7 (IQR 0.5)	Aldosterone: Aldosterone Coat-A-Count, pmol/L Renin: Cis-Bio Renin III (International France) direct method, mU/L	36/200 (18.0%) screened positive 27/36 (75.0%) proceeded to confirmatory testing 11/27 (40.7%) tested positive on confirmatory test	One or two ARR > 65 pmol/mU	FST. Post-FST PAC threshold 225 pmol/L based on a previous study^31^	Prevalence, 5.5% (11/200) with incomplete PAC suppression post-FST
7	Schmiemann (2012) [16] Prospective Germany	BP > 140/90 mmHg while on ≥3 antihypertensives 2 urban group practices in Northern Germany	IC: BP > 140/90 mmHg while on ≥3 antihypertensives EC: Pregnancy, known secondary hypertension, necessity of spironolactone	*N* = 63 30%M, 70%F Mean 69 (SD 10.4)	ARR after cessation of beta-blockers for 2 weeks (4 weeks for spironolactone)	Pre-existing hypoK corrected	Aldosterone: RIA, pg/mL Renin: RIA, pg/mL	15/63 (23.8%) screened positive 3/15 (20.0%) proceeded to confirmatory testing 3/3 (100%) tested positive on confirmatory test	ARR > 45 pg/mL:pg/mL and PAC > 310 pg/mL	“Sodium chloride loading test” and/or diagnostic imaging It is unclear if the “sodium chloride loading test” is SIT or OSLT	Prevalence, 4.8% (3/63) 2 APA, 1 IHA
8	Westerdahl (2006) [13] Prospective Sweden	Hypertension	IC: 75 years of age or younger EC: insulin-dependent diabetes mellitus, dementia, stroke, mental health disorder, malignant tumours	*N* = 200 Sex breakdown not stated Mean age not stated	Beta blockers, ACE-I, alpha-1 receptor blocking, angiotensin II-antagonists withdrawn 2 weeks before CCBs continued	ARR > 100 pmol/L per ng/L: 3.9 (0.3) ARR < 100 pmol/L per ng/L: 4.0 (0.2)	Aldosterone: RIA DPC Skafte AB, pmol/L Renin: Pasteur method (Elektrabox), ng/L	50/200 (25.0%) screened positive 26/50 (52.0%) proceeded to confirmatory testing 16/26 (61.5%) tested positive on confirmatory test	ARR > 100 pmol/L per ng/L Confirmed by in-house ARR (age 21–57 years, 11 men, 17 women) and derived upper limit by mean + 2 SD	FST. Post-FST PAC > 160 pmol/L on day 5, in sitting position after 15-min rest	Prevalence, 8.5% (17/200) FST: 16/26 had incomplete PAC suppression; 1 had previously diagnosed PA
9	Loh (2000) [17] Prospective Singapore	Hypertension 2 large primary care clinics	IC: Not stated EC: Renal impairment (serum creatinine >140 µmol/L), treatment with spironolactone or glucocorticoids	*N* = 350 All: 39%M, 61%F PA: 56%M, 44%F EH: 38%M, 62%F All: 55.2 years (SD 8.6) PA: 50.6 years (11.3) EH: 55.4 years (8.6)	No description of withholding or adjusting medications	PA: 3.7 (0.1) EH: 4.2 (0.0)	Aldosterone: Coat-A-Count ALD RIA (Diagnostic Products) Renin: PRA GammaCoat PRA RIA (INCSTAR Corp)	63/350 (18.0%) screened positive 56/63 (88.9%) proceeded to confirmatory testing 16/56 (28.6%) tested positive on confirmatory test	ARR > 20 ng/dL:ng/ml/h and PAC > 15 ng/dL (>416 pmol/L) based on 95th percentile ARR and 75th percentile PAC derived from healthy normotensive volunteers	Potassium supplementation if hypokalaemia SIT (seated). PA if post-SIT PAC failed to suppress to <10 ng/dL (<277 pmol/L)	Estimated prevalence, 5.1% Computed tomography and adrenal vein sampling: 8 potentially curable by unilateral adrenalectomy

*BP* blood pressure, *SBP* systolic blood pressure, *DBP* diastolic blood pressure, *MRA* mineralocorticoid receptor antagonist, *PA* primary aldosteronism, *APA* aldosterone-producing adenoma, *BAH* bilateral adrenal hyperplasia, *PRA* plasma renin activity, *PRC* plasma renin concentration, *PAC* plasma aldosterone concentration, *ARR* aldosterone-to-renin ratio, *RIA* radioimmunoassay, *OSLT* oral salt loading test, *SIT* saline infusion test, *FST* fludrocortisone suppression test, *CCT* captopril challenge test, *SD* standard deviation, *IC* inclusion criteria, *EC* exclusion criteria, *M* male, *F* female, *Na*, sodium, *K* potassium, *HypoK* hypokalaemia, *USA* United States of America, *U* urinary, *ACE-I* angiotensin-converting enzyme inhibitor

^a^Protocol violation: four patients had ongoing amiloride (5 mg/day) and normal ARR. They were not excluded from study as unlikely to affect overall results though there is risk of false negative)

^b^Chosen ARR cut-off half of that used in clinical practice and close to mean for healthy Swedish population

The studies incorporated heterogenous ARR thresholds across heterogenous study populations, making direct head-to-head comparisons challenging. In the studies where PRA was measured (*n* = 3), two studies used an ARR threshold of ≥20 ng/dL:ng/mL/h [10, 17] together with a PAC threshold of ≥10 ng/dL (and suppressed PRA (<1 ng/mL/h) in one study [10] and PAC threshold of >15 ng/dL in the other [17]. The third study set a higher ARR threshold of >30 ng/dL:ng/mL/h and required a PAC > 10 ng/dL for an abnormal screening test [18]. In studies that measured PRC (*n* = 6), the ARR threshold ranged from >31 to >78 pmol/mU with four of the six studies also requiring an elevated PAC varying from >277 pmol/L to >859 pmol/L [11–16]. Based on the range of ARR thresholds, the true positive rates ranged from 12.2% to 65.4% while the false positive rates ranged from 34.6% to 87.8% (Table 2). At the lowest ARR threshold of >31 pmol/mU (with a PAC threshold of >350 pmol/L), 33.3% of subjects were confirmed to have PA [11]; at the highest ARR threshold of ≥100 pmol/mU (with a PAC threshold of >277 pmol/L), 42.7% of patients were confirmed to have PA [18].

**Table 2:** True and false positive rates for different plasma aldosterone-to-renin ratio thresholds

Study	True Positive ARR	False Positive ARR	Population	ARR Threshold (pmol/mU)	ARR Threshold (pmol/ng)	ARR Threshold (ng/mU)	ARR Threshold (ng/dL:ng/ml/h)
Galati (2016) [10]	2/6 (33.3%)	4/6 (66.7%)	Hypertension	ARR ≥ 67 pmol/mU with PAC ≥ 277 pmol/L and suppressed PRC (<8.2 mU/L)	ARR > 108 pmol/ng with PAC ≥ 277 pmol/L and suppressed PRC (<8.2 mU/L)	ARR > 24 ng/mU	*ARR ≥ **20** **ng/dL per ng/ml/h with PAC** ≥ 10 ng/dL and suppressed PRA (<**1** **ng/mL/h)**
Volpe (2013) [11]	2/6 (33.3%)	4/6 (66.7%)	Hypertension	ARR > 31 pmol/mU and PAC > 350 pmol/L	*ARR > **50 pmol/ng and PAC** > **350 pmol/L**	ARR > 11 ng/mU and PAC >13 ng/dL	ARR > 9 ng/dL:ng/ml/h and PAC > 13 ng/dL
Xu (2020) [14]	40/93 (43.0%)	53/93 (57.0%)	Hypertension	*ARR > **55 pmol/mU and PAC** > **277 pmol/L**	ARR > 89 pmol/ng and PAC > 277 pmol/L	*ARR > **20** **ng/mU and PAC** > **10** **ng/dL**	ARR > 16 ng/dL:ng/ml/h and PAC > 10 ng/dL
Kayser (2018) [15]	9/74 (12.2%)	65/74 (87.8%)	Hypertension	*ARR > **40 pmol/mU and PAC** > **400 pmol/L**	ARR > 65 pmol/ng and PAC > 400 pmol/L	ARR > 14 ng/mU and PAC > 15 ng/dL	ARR > 12 ng/dL:ng/ml/h and PAC > 15 ng/dL
Monticone (2017) [18]	99/232 (42.7%)	133/232 (57.3%)	Hypertension	ARR ≥ 100 pmol/mU and PAC > 277 pmol/L	ARR ≥ 161 pmol/ng and PAC > 277 pmol/L	ARR ≥ 35 ng/mU and PAC > 10 ng/dL	*ARR > **30** **ng/dL per ng/ml/h (>830 pmol/L per ng/mL/h) and PAC** > **10** **ng/dL (>277 pmol/L)**
Westerdahl (2011) [12]	11/32 (34.4%)^a^	21/32 (65.6%)^a^	Hypertension	*ARR > **65 pmol/mU**	ARR > 105 pmol/ng	ARR > 23 ng/mU	ARR > 19 ng/dL:ng/ml/h
Schmiemann (2012) [16]	3/15 (20.0%)	12/15 (80.0%)	Resistant hypertension	ARR > 78 pmol/mU and PAC > 859 pmol/L	ARR > 126 pmol/ng and PAC > 859 pmol/L	ARR > 28 ng/mU and PAC > 310 pg/mL (note: unit for PAC is pg/mL)	*ARR > **45** **pg/mL per pg/mL and PAC** > **310** **pg/mL** (note: unit for ARR is pg/mL per pg/mL)
Westerdahl (2006) [13]	17/26 (65.4%)	9/26 (34.6%)	Hypertension	ARR > 63 pmol/mU	*ARR > **100 pmol/ng**	ARR > 20 ng/mU	ARR > 18 ng/dL:ng/ml/h
Loh (2000) [17]	16/56 (28.6%)	40/56 (71.4%)	Hypertension	ARR > 67 pmol/mU and PAC > 416 pmol/L	ARR > 108 pmol/ng and PAC > 416 pmol/L	ARR > 20 ng/mU and PAC > 15 ng/dL	*ARR > **20** **ng/dL per ng/ml/h and PAC** > **15** **ng/dL**

*ARR* aldosterone-to-renin ratio, *PRA* plasma renin activity, *PRC* plasma renin concentration, *PAC* plasma aldosterone concentration

ARR and aldosterone concentrations have been converted to several common units of measurement for ease of comparison. The units reported in the original studies are indicated by an asterisk (*) and highlighted bold. PRA of 1 ng/mL/hr is assumed to be approximately PRC of 8.2 mU/L

^a^Thirty six patients had raised ARR on initial testing. However, four patients abstained from further testing and they were excluded from the calculation of true and positive ARR rates

Seven of the nine studies required only one positive ARR before proceeding to confirmatory testing [10, 13–18]. However, in the study by Volpe and colleagues, if the initial ARR was elevated, a repeat ARR (and a 24-hour urinary aldosterone with sodium and potassium) was required prior to any confirmatory testing [11]. At the initial screening an elevated ARR was found in 14 patients. After adjustment of medication and repeated ARR with urinary aldosterone, six patients were considered to have positive screening tests [11]. The repeat ARR led to fewer than half (43%) of participants proceeding to confirmatory testing. In the study by Westerdahl and colleagues, patients with one or two high ARR (>65 pmol/mU) at screening were referred for FST. Of the 36 patients with raised ARR on initial testing, the second ARR led to three less patients proceeding to confirmatory testing [12]. All studies included in our review used an immunoassay for aldosterone and renin measurements but a variety of different analysers were used (Table).

Six studies incorporated either oral salt loading, saline infusion, fludrocortisone suppression or captopril challenge test to confirm the diagnosis of PA [10, 12, 13, 15–17]. Three studies allowed two or more confirmatory tests to be used within its study population [11, 14, 18].

## Discussion

This review demonstrated substantial heterogeneity in the cut-off values for the ARR that are used to determine if the hypertensive patient should undergo further testing for PA. There was also variability in the requirement for an absolute minimum PAC in the screening process.

The heterogeneity in ARR thresholds can be partly attributed to the lack of uniform guideline recommendations. Of note, rather than a definitive threshold, suggested ranges for the ARR cut-off, without an absolute minimum PAC threshold, are provided in the Endocrine Society Guidelines [4]. Furthermore, for the majority of the studies included in our review, the derivation of the ARR and/or PAC thresholds were not clearly described (Supplementary Table 1). Some studies adopted a lower cut-off compared to previously published studies to avoid false negatives [10, 11, 15]. Loh and colleagues established their own threshold using 150 healthy volunteers with normal blood pressure and normokalaemia [17]. Westerdahl and colleagues verified the ARR threshold by determining the +2 SD from the mean of 28 healthy subjects [13]. As expected, the higher the ARR and absolute minimum PAC threshold, the greater proportion of patients are confirmed to have PA. At an ARR of ≥100 pmol/mU and a PAC of >277 pmol/L, 42.7% of the screening results were true positives [18]. In comparison, only 12.2% of the screening results were considered to be true positives with an ARR of >40 pmol/mU and PAC > 400 pmol/L [15]. On the other hand, an ARR threshold of >65 pmol/mU, without a minimum PAC requirement, represented a true positive result in 30.6% of patients [12]. However, different confirmatory tests with their own variability in diagnostic accuracy were used to define the true positives in these studies, which prevents generalisation about the most appropriate ARR threshold [19].

Between-assay variability is another reason for different recommended ARR thresholds. All of the ARRs in this review were measured by immunoassays. The possibility of between-method differences in ARR immunoassay measurements cannot be excluded. Fortunato and colleagues compared the analytical performance of two CLIAs (IDS iSYS and Diasorin LIAISON) and some RIA methods [20]. The aldosterone values measured with the LIAISON platform were compared to those measured with the iSYS platform in 290 plasma samples of 91 healthy subjects and 199 patients with cardiovascular diseases [20]. There was a significant bias (*P* = 0.0146) between these two methods, which proportionally increased with the aldosterone concentration [20]. Compared to RIA methods, the LIAISON method showed on average lower aldosterone values of about −11.2% (SD 118.2%, *P* < 0.0001 by Wilcoxon Signed Rank test) [20]. Between the ALDOCTK-2 RIA and iSYS platform, the mean difference was 40.7 pmol/L with the range between ±1.96 SD from −38.0 to 119.4 pmol/L [20]. Similar variability has been reported between other immunoassay methods [21, 22].

Developments in LC–MS/MS have allowed aldosterone to be quantified in a more consistent and accurate manner in routine clinical laboratories [23]. The LC–MS/MS system will likely lead to lower ARR and/or PAC thresholds compared to immunoassay. Guo and colleagues compared aldosterone measurement by RIA with LC–MS/MS (PRC by immunoassay) in 41 patients who underwent ARR testing to screen for, and FST to confirm or exclude, PA [24]. The median serum PAC with LC–MS/MS was 27.8% lower (*P* < 0.05) than plasma PAC by RIA in 164 pairs of FST samples [24]. A cut-off of 55 pmol/mU for LC–MS/MS PAC-based ARR was equivalent to the threshold of 70 pmol/mU for RIA PAC-based ARR [24].

An important clinical question that requires further investigation is within-individual variability in the ARR and whether a single ARR at one point in time is sufficient to exclude PA or indicates the need for confirmatory testing. Yozamp and colleagues found that aldosterone concentrations and the ARRs are highly variable in patients with PA, with many screening values falling below conventionally accepted thresholds [25]. Based on 51 patients with confirmed PA who had two or more screening aldosterone and renin measurements on different days, the within-individual variability was 31% for aldosterone and 45% for ARR [25]. Of note, 57% of subjects had at least one ARR below 30 ng/dL:ng/mL/h, 27% had at least two ratios below 30 ng/dL:ng/mL/h, and 24% had at least one ARR below 20 ng/dL:ng/mL/h [25]. Only two of the studies in our current review required more than one ARR result for a ‘positive’ screen. The ARR variability may also lead to an initially negative ARR despite the presence of PA. Patients in this category would have been missed in the studies included in our review.

A limitation of the ARR is that in the presence of very low renin levels, the ARR may be elevated even when plasma aldosterone is low and almost certainly not consistent with PA [4, 26]. To avoid this problem, some investigators include a minimum PAC threshold, ranging from >277 pmol/L (>10 ng/dL) to >859 pmol/L (>31 ng/dL), within the screening criteria [26]. Some proceed with a diagnostic workup for PA in all patients with elevated ARR unless the PAC is below the level used to define normal suppression during confirmatory testing [4, 26]. Having a minimum PAC threshold has previously been shown to reduce the sensitivity of the ARR [27], but demonstrated inconsistent effect on the proportion of true positives in the studies reviewed here.

The ARR can be significantly influenced by commonly used antihypertensive medications. These medications affect either aldosterone or renin concentration, and should be considered when interpreting the ARR as the accuracy of screening may be undermined [1, 6]. The lack of consistent control for these medications in the studies included in our review may also contribute to heterogeneity in the diagnostic performance of the ARR. Interfering medications should be ceased and replaced with sustained-release verapamil, prazosin, moxonidine and/or hydralazine [6]. For accurate screening, drugs that should be ceased for at least four weeks before the test include thiazide diuretics, loop diuretics, mineralocorticoid receptor antagonists, epithelial sodium channel blockers. Where possible, medications that should be ceased for at least two weeks before test are angiotensin receptor blockers, angiotensin-converting enzyme inhibitors, dihydropyridine calcium channel blockers, selective and non-selective beta blockers [6].

The lack of standardisation in testing conditions may have also contributed to the seemingly disappointing performance of the ARR [28]. The challenges in setting the ‘optimal’ threshold is another contributing factor. Adjusting threshold for any diagnostic test can affect a test’s sensitivity and specificity [29]. This is simply a mathematical function and not specific to PA. It is at the discretion of the clinician to select the threshold to be used based on their priorities between sensitivity and specificity because there will be inherent trade-offs between the two. This “moving threshold” approach to ARR has not been used in many studies pertaining to ARR interpretation and there is inherent fallibility of a single ARR threshold for a disease (such as PA) that exists on a biochemical continuum [29].

A limitation of any analysis of diagnostic accuracy is the lack of gold standard and this is relevant to the ARR and PA diagnosis. The only gold standard available is for patients with unilateral PA who achieve a biochemical cure defined by the normalisation of the ARR and potassium following adrenalectomy [30]. Of the 10 studies in our review, one study conducted a subgroup analysis of unilateral PA [14]. Xu and colleagues reported that for surgically treated patients (*n* = 7), a complete biochemical success rate was 100% and a complete clinical success rate (defined by the normalisation of blood pressure without any medications) was 85.7% [14]. Based on their ARR threshold of >20 ng/dL:mU/L (>55 pmol/mU) and a PAC of >10 ng/dL (>277 pmol/L), 7.5% (7/93) were confirmed to have unilateral disease [14].

Of all the studies in this review, none conducted confirmatory testing in subjects with a ‘negative’ ARR. Hence, we could not reliably determine an overall ARR sensitivity and specificity, nor can we recommend the adoption of any single threshold for ARR interpretation.

In contrast to the studies included in our review where the ARR was routinely performed, recent studies in large hypertensive cohorts revealed much lower prevalence of PA due to the lack of systematic screening. In a nationally distributed cohort of veterans in the US with apparent treatment-resistant hypertension (*n* = 269,010), Cohen and colleagues observed that testing for PA was rare; fewer than 2% of patients with incident treatment-resistant hypertension underwent guideline-recommended testing for PA [31]. Testing rates ranged from 0% to 6% across medical centres and did not correlate to the population size of patients with apparent treatment-resistant hypertension [31]. Liu and colleagues found in a Canadian study of 1.1 million adults with hypertension that less than 1% of patients expected to have PA were ever formally diagnosed and treated [32]. Of note, among those who were screened, 1703 (21.4%) had positive test results consistent with possible PA, and 1005 (59.0%) of these were further investigated to distinguish between unilateral and bilateral forms of PA [32]. Despite it being an imperfect first-line test for detecting PA (affected by many common medications, time of day, posture, stage of menstrual cycle, and renal impairment), the ARR test may still be valuable for excluding primary aldosteronism when two ARR results on different days are negative and for identifying potential primary aldosteronism where there is a positive ARR or low plasma renin, especially if the patient is taking an ACE inhibitor, angiotensin receptor blocker, or diuretic that should increase renin [2, 4]. When the ARR test is positive, referral to a specialist unit for confirmatory primary aldosteronism testing is recommended [4].

## Conclusion

A range of ARR thresholds are used for the screening of PA around the world. Periodic clinical audits and case reviews by clinicians and the laboratory may help refine the local thresholds. In practice, the GP who is ordering the test can rely on the cut-off or decision limit recommended by their local pathology service. The patients with an abnormally elevated ARR should be referred to a specialist for confirmatory testing while patients with a normal ARR could have a repeat test in view of the within-individual variability. If the ARR threshold used in clinical practice is too low, the next confirmatory step should help to identify those who have a false positive ARR and spare these patients from further testing. If the ARR threshold is set too high, then a patient with PA may miss out on the accurate diagnosis and targeted treatment of PA.

## Supplementary Information

Supplementary Table 1 ARR Primay Care
