# Supplementary material for: Performance of the aldosterone-to-renin ratio as a screening test for primary aldosteronism in primary care
Source: Endocrine. 2022 May 27;77(1):11–20. doi: 10.1007/s12020-022-03084-x (PMC9242901; doi:10.1007/s12020-022-03084-x)
Supplement: Supplementary file 1 — Supplementary Table 1 ARR Primay Care [file 12020_2022_3084_MOESM1_ESM.docx]

**Supplementary Table 1. Sources of the plasma aldosterone-to-renin ratio and/or plasma aldosterone concentration thresholds for the screening of primary aldosteronism used in the studies**

ARR, plasma aldosterone-to-renin ratio; PAC, plasma aldosterone concentration; PRC, plasma renin activity; PA, plasma aldosteronism

| **Study** | **Source of ARR and/or PAC thresholds** |
| --- | --- |
| Loh et al (2000)[1] | - Reference intervals for PAC, PRA and ARR were established using 150 healthy volunteers aged 19-65 years who were normotensive and normokalaemic. A positive screening test for PA was defined arbitrarily based on the 95^th^ percentile ARR and the 75^th^ percentile PAC |
| Westerdahl et al (2006)[2] | - The source of the ARR threshold was a study by Racine and colleagues[3]. The threshold was verified by testing 28 healthy subjects (11 men and 17 women, aged 21-57 years) and the upper limit for ARR determined by the mean value for the ratio +2 SD |
| Westerdahl et al (2011)[4] | - The sources of ARR or PAC threshold were not described but stated that “it is still possible that the cut-off value for ARR was too high in this context, with the potential consequence that mild forms of PA may not have been detected” |
| Schmiemann et al (2012)[5] | - The ARR reference was according to the local laboratory |
| Volpe et al (2013)[6] | - The ARR threshold was “half of that used in clinical practice” and “close to the mean for the healthy Swedish population”[2] - The PAC threshold was a “hypothetical” limit |
| Galati et al (2016)[7] | - The source of the ARR threshold was not described. Compared to other studies, a lower ARR threshold was set to reduce the chance of false negatives - The source of the PAC threshold was not stated |
| Monticone et al (2017)[8] | - The sources of ARR and PAC thresholds were not described |
| Kayser et al (2018)[9] | - The source of the ARR threshold was not discussed. Compared to previous studies, a lower ARR threshold was used to miss as few PA patients as possible - The source of the PAC threshold was not stated. The use of PAC threshold was to prevent too many false-positive test results |
| Xu et al (2020)[10] | - The sources of ARR and PAC thresholds were not described. But the authors cited Westerdahl et al[4] and Omura et al[11] as two studies “with a similar ARR cut-off” |

**References**

1 Loh, K. C., Koay, E. S., Khaw, M. C., Emmanuel, S. C. & Young, W. F., Jr. Prevalence of primary aldosteronism among Asian hypertensive patients in Singapore. J Clin Endocrinol Metab **85**, 2854-2859 (2000).

2 Westerdahl, C., Bergenfelz, A., Isaksson, A., Wihl, A., Nerbrand, C. & Valdemarsson, S. High frequency of primary hyperaldosteronism among hypertensive patients from a primary care area in Sweden. Scand J Prim Health Care **24**, 154-159 (2006).

3 Racine, M. C., Douville, P. & Lebel, M. Functional tests for primary aldosteronism: value of captopril suppression. Curr Hypertens Rep **4**, 245-249 (2002).

4 Westerdahl, C., Bergenfelz, A., Isaksson, A., Nerbrand, C. & Valdemarsson, S. Primary aldosteronism among newly diagnosed and untreated hypertensive patients in a Swedish primary care area. Scand J Prim Health Care **29**, 57-62 (2011).

5 Schmiemann, G., Gebhardt, K., Hummers-Pradier, E. & Egidi, G. Prevalence of hyperaldosteronism in primary care patients with resistant hypertension. J Am Board Fam Med **25**, 98-103 (2012).

6 Volpe, C., Wahrenberg, H., Hamberger, B. & Thorén, M. Screening for primary aldosteronism in a primary care unit. J Renin Angiotensin Aldosterone Syst **14**, 212-219 (2013).

7 Galati, S. J. *et al.* PREVELENCE OF PRIMARY ALDOSTERONISM IN AN URBAN HYPERTENSIVE POPULATION. Endocr Pract **22**, 1296-1302 (2016).

8 Monticone, S. *et al.* Prevalence and Clinical Manifestations of Primary Aldosteronism Encountered in Primary Care Practice. J Am Coll Cardiol **69**, 1811-1820 (2017).

9 Käyser, S. C., Deinum, J., de Grauw, W. J., Schalk, B. W., Bor, H. J., Lenders, J. W., Schermer, T. R. & Biermans, M. C. Prevalence of primary aldosteronism in primary care: a cross-sectional study. Br J Gen Pract **68**, e114-e122 (2018).

10 Xu, Z. *et al.* Primary Aldosteronism in Patients in China With Recently Detected Hypertension. J Am Coll Cardiol **75**, 1913-1922 (2020).

11 Omura, M., Saito, J., Yamaguchi, K., Kakuta, Y. & Nishikawa, T. Prospective study on the prevalence of secondary hypertension among hypertensive patients visiting a general outpatient clinic in Japan. Hypertens Res **27**, 193-202 (2004).
